# Supplementary material for: How does the online innovation community climate affect the user’s value co-creation behavior: The mediating role of motivation
Source: PLoS One. 2024 Apr 23;19(4):e0301299. doi: 10.1371/journal.pone.0301299 (PMC11037537; doi:10.1371/journal.pone.0301299)
Supplement: Attachment — Submitted filename: Response to Reviewers.docx [file pone.0301299.s002.docx]

Dear editor,

On behalf of my co-authors, we thank you very much for your letter and for the reviewers’ comments concerning our manuscript entitled “How does the online innovation community climate affect the user’s value co-creation behavior: the mediating role of motivation”. These comments are all valuable and helpful for revising and improving our papers, as well as the important guiding significance to our research. We have studied your comments carefully and have made corrections which we hope meet with approval. The main corrections in the paper and the responds to your letter and the reviewers’ comments are as following. We marked the revised places in red.

**<1> To editor:**

1. Please ensure that your manuscript meets PLOS ONE's style requirements, including those for file naming. The PLOS ONE style templates can be found at 
https://journals.plos.org/plosone/s/file?id=wjVg/PLOSOne_formatting_sample_main_body.pdf and 
https://journals.plos.org/plosone/s/file?id=ba62/PLOSOne_formatting_sample_title_authors_affiliations.pdf

**Reply:** We have changed the format according to the PLOS ONE style templates, and hope it will meet PLOS ONE’s style requirements.

2. We note that the grant information you provided in the ‘Funding Information’ and ‘Financial Disclosure’ sections do not match.

When you resubmit, please ensure that you provide the correct grant numbers for the awards you received for your study in the ‘Funding Information’ section.

**Reply:** We have added and revised the grant numbers in ‘Funding Information’ section.

3. Thank you for stating the following financial disclosure: "This work was supported by the National Social Sciences Foundation of China (Project No. 18CSH019), the Social Sciences Foundation of Beijing (Project No. 20JCC096), and Beijing Municipal Education Commission (Project No. SM201910011007)."

Please state what role the funders took in the study.  If the funders had no role, please state: "The funders had no role in study design, data collection and analysis, decision to publish, or preparation of the manuscript."

If this statement is not correct you must amend it as needed.
 
Please include this amended Role of Funder statement in your cover letter; we will change the online submission form on your behalf.

**Reply:** Thank you very much for your guidance on the “financial disclosure”. We have added the founder role of Funder statement in the cover letter.

4. In your Data Availability statement, you have not specified where the minimal data set underlying the results described in your manuscript can be found. PLOS defines a study's minimal data set as the underlying data used to reach the conclusions drawn in the manuscript and any additional data required to replicate the reported study findings in their entirety. All PLOS journals require that the minimal data set be made fully available. For more information about our data policy, please see http://journals.plos.org/plosone/s/data-availability.

**Reply:** We have uploaded the data via "Supporting Information". Please let us know if we do it wrong and we will revise the Data Availability according your instruction.

**<2> To Reviewer 1:**

1.The version of the abstract is not good, it is recommended that the author can add background and value section, based on the structured abstract.

**Reply**:

Thank you very much for your guidance on the Abstract. We have added the background information of the OIC and the value section. The revised abstract is as follow:

Online Innovation Community (OIC) serves as a virtual space for users to exchange products and services, and share knowledge and information. Previous studies have indicated that community climate is an important factor affecting users' value co-creation behavior, however, the influencing process has not been clearly revealed from the perspective of motivation. In this study, we explored the relationship between online innovation community climate (supportive climate and controlling climate), user motivation and value co-creation behavior (user’s participation behavior and user’s citizenship behavior) based on the SOR model. This study sample included 29,835 pieces of information from 3,315 users in 14 product sections of the OnePlus Community which were analyzed with Mplus8.1. The findings revealed that: (1) The supportive climate had a positive impact on user’s citizenship behavior(β=0.042), while the controlling climate exerted a significant positive impact on user’s citizenship behavior (β=0.078) and user’s participation behavior(β=0.099); (2) The need for achievement played a suppressing effect between community climate and user’s participation behavior, the need for power played a suppressing effect between supportive climate and user’s value co-creation behavior, and the need for affiliation played a mediating role between supportive climate and user’s citizenship behavior (β=0.010) and user’s participation behavior(β=0.006); (3) Community trust positively moderated the relationship between the need for achievement and user’s participation behavior(β=0.058) as well as between the need for power and user’s participation behavior(β=0.043).

2. In the Keywords, they are just so-so and the authors keep the order from the capital letter "A-Z". Besides, the number of keywords can be reduced to 5-6 and divided them by semicolon.

**Reply:**

We have changed the order by "A-Z" and revised the number of keywords are 5 as you suggested. The revised Keywords are as follow:

Keywords: community climate; motivation; online community; trust; value co-creation

3. In the Introduction, the materials of the paper should be just update to 2022, and the authors also can look through the recent and important paper on this topic again. It lacks important literature. In the Literature section, we should link to the recent and most literature, specially 2020-2023. Besides, we also pay more attention to the value and contribution of the paper. The authors should add it, use literature to develop the arguments. The literature should be updated, the following can be cited, I also suggest that.

DOI:10.1108/IJIS-12-2022-0237
DOI: 10.1016/j.heliyon.2023.e13192

**Reply:**

There are several recently published papers we have read, some of which published in high impact journals. We have cited these related literatures in line36, line63 and line73.

4.In the literature review, the literature can be updated to the year of 2023. Besides, the literature upon the topic “innovative behavior” can cite the following, I suggest that

DOI:10.1007/s12144-020-01120-5
DOI:10.2147/PRBM.S380989
DOI:10.1108/BJM-09-2019-0335

**Reply:**

Thank you very much for the suggestion about the literature review, we have cited these important literatures in line97, line114, Line128, line147, line193 and line224.

5.In the section of methodology including selecting data, the authors should add more details into the section as much as possible.

**Reply**:

We have added some information about the OnePlus mobile community and the operational measures. The revised contents were:

This study analyzed the OnePlus Community ([https://www.oneplusbbs.com](https://www.oneplusbbs.com/)), established in 2013 by OnePlus mobile phone officials. It is a virtual community where users can exchange products and services, as well as share knowledge and information. In OnePlus Community, users have opportunities to express their views and share their experiences. According to the report by Counterpoint in the first half of 2023, OnePlus was recognized as one of the top ten brands with the fastest growth in global market share and it had more than 15 million community users in 196 countries around the world. The users in OnePlus Community are highly active, the rich user resources owned by the community contribute to this research work.

In order to collect user data, members of the research group registered as community users and gathered information from OnePlus Community Forum, including the number of new posts, the number of comments, and the number of posts selected as "hot". As required by the community, users can only discuss product experience and improvement suggestions in the corresponding product section. To collect users’ product experience properly, we sampled 14 product sections in the community by Python (as of data collection, a total of 17 sections, excluding three sections beyond the research purpose, including Youleyuan, Qingsheying and Official activities) as the primary source for information collection. Since only the latest 1,000 pages of posts and related information (19 posts per page, 266,000 posts in total) from the time of data capture were available on the website of the target community, we were unable to obtain all the posts of the community since its establishment. Still, the crawled data were sufficient to meet our research needs, and the data posted up to October 4, 2021 were collected.

The data required for the research involves both users and products, which were collected in three rounds: in the first round, posts that were officially rated as “hot” in the community were collected, as shown in Figure 2, including the popularity value, product section where the post was located, and the link to the personal website. A total of 38,894 posts were collected. In the second round, leveraging the uniqueness of each user's personal website has been obtained in the first round, basic user information was collected, including user's name, ID, number of cheers, displayed items of user information, online time, number of friends, etc. In the third round, the number of comments deleted by the authority, the number of comments on the post, and the number of views on the post collected in the first round were collected. Finally, a total of 676,380 pieces of information were collected.

The inclusion and exclusion criteria are as follows: (1) official activity posts; (2) posts and user information with incomplete information; and (3) only one post randomly included for the same user. As the collection of user information data in this study was related to the posts by users, if all posts by the same user were included, the same user information would be collected repeatedly for users who post multiple times, which would cause problems for data cleaning and sorting. In addition, we randomly deleted multiple posts from the same user to ensure the accuracy of the results. Totally 29,835 pieces of information from 3,315 users were finally included for analysis.

6.The authors should make the definitions of the variables clearly in the section of “variables”.

**Reply:**

We have added the definitions of the variables, the revised contents were as follows:

(1) Dependent variable: User participation behavior (UPB) refers to the behavior that users actively participate in value-creating activities in the community, so it was measured by the number of posts by users[12]； user citizenship behavior (UCB) refers to the behavior that users spontaneously safeguard users' interests and create additional value in the community, which is measured by the number of comments posted by users [12].
(2) Independent variable: Supportive climate (SC) refers to the climate in which users are encouraged to post freely, communicate on an equal footing and develop friendly relations in the community [16], so it was measured by the hot value of posts marked by the community. Controlling climate (CC) refers to the flow of information in the community, the words, deeds and behaviors of members were restricted [16], so it was measured by the number of comments on posts that were officially deleted.
(3) Mediating variable: The need for achievement (NA) refers to the need of users to achieve goals and pursue excellence based on their own interests and passions [43], so it was represented by the number of cheers users get in the OnePlus Community [56]. The need for power (NP) refers to the user’s need to influence or control others by acquiring social status [56], so it was indicated by the different user group levels that users belong to in the community. There are 11 user groups in the OnePlus Community, and different user group levels are set according to the behaviors of users participating in community activities. Users with stronger community power have a wider scope of activities and comment permissions than users with weaker power. The higher the user group level is, the stronger the influence on others is. The need for affiliation (NFA) refers to the need of users to pursue friendliness and value interpersonal relationships [56], so it was measured by the number of friends a user has in the community.
(4) Moderating variable: Community trust (TR) refers to the user’s trust in the community [52]. In this study, we argue that users think they belong to a certain group. The higher the identification with the community is, the more they believe that the community can represent their own image, the more they trust the community, the more likely they are to display their personal information in the community. Therefore, community trust was measured by items in which users display personal information in the community.
(5) Control variable: User age in community (UAC): UAC can have a significant impact on users’ participation in community activities [58]. In this study, it was represented by the time between user registration and data collection. Product section in community (PSC): the OnePlus Community has set up multiple product sections according to product types and stipulates that users must post content under the corresponding section; otherwise, the post will be deleted. For users, they often have rich experience in using the products they purchased, so they are more likely to share their experience in a fixed product section instead of participating in the activities of other product sections. For this reason, we used PSC as a control variable, which was indicated by the product section to which the user’s content belongs.

7. The authors should control the number of table and some of them can be integrated.

**Reply:**

We have integrated the table of the moderating role of the community trust between the need for achievement and the user’s participation behavior, the need for power and the user’s participation behavior, to make the results more clearly. The revised contents were:

We employed hierarchical regression to test the moderating effect of community trust. Since the need for achievement did not have a significant impact on user’s citizenship behavior, only the mediating role of community trust between the need for achievement and user’s participation behavior, and the need for power and affiliation on user’s value co-creation was analyzed. To avoid collinearity, the variables of the need for power and community trust were centralized before testing the mediating effect, and then the interaction item was calculated. Given the length constraints of the article, the valid results of the hypotheses were presented in Table6.

Table 6: The test results of the moderating effect

| Variable | Participation behavior | | | Participation behavior | | |
| --- | --- | --- | --- | --- | --- | --- |
|  | Model 1 | Model 2 | Model 3 | Model 4 | Model 5 | Model 6 |
| UAC | 0.220*** | 0.260*** | 0.264*** | 0.220*** | 0.011 | 0.012 |
| PSC | -0.015 | 0.015 | 0.024 | -0.015 | -0.027 | -0.027 |
| NA |  | -0.128*** | -0.142*** |  |  |  |
| NP |  |  |  |  | 0.413*** | 0.417*** |
| TR |  | 0.143*** | 0.125*** |  | 0.062*** | 0.056** |
| Interaction item |  |  | 0.058** |  |  | 0.044** |
| F | 94.066 | 39.087 | 9.355 | 94.066 | 267.109 | 7.510 |
| R^2^ | 0.054 | 0.076 | 0.078 | 0.054 | 0.185 | 0.186 |
| ΔR^2^ | 0.054 | 0.022 | 0.003 | 0.054 | 0.131 | 0.002 |

In Model 3 of Table 6, it can be seen that the interaction term between the need for achievement and community trust had a positive impact on user participation behavior (β = 0.058, p < 0.01), and H6a was validated. Additionally, the interaction term between the need for power and community trust exhibited a positive impact on user participation behavior (β = 0.044, p < 0.01), and H6c was validated. However, other hypotheses were not validated.

8. Before the conclusion section, the authors should add discussion section, comparing the findings of the study with previous studies. However, it is missing.

**Reply:**

We are honored to receive the comments and guidance about the discussion, we integrated the conclusion and discussion to make the results more clarify. The revised discussion was:

This study explored the impact of the online community climate on users’ value co-creation behavior in the OnePlus Community and analyzed the mediating role of motivation and the moderating role of community trust. The findings are as follows:

(1) Supportive climate positively affected user’s citizenship behavior, and controlling climate positively affected user’s participation behavior and user’s citizenship behavior. The result is in line with the findings of Zhao et al. [6], whose study showed that supportive climate can promote users' value co-creation behavior by enhancing their psychological capital. Zhu’s (2020) [33] study showed that controlling climate can promote users’ innovation behavior. The stronger the supportive and controlling climate provided by the community, the more it can promote users' information exchange and resource acquisition behavior in the community, thus promoting their value co-creation behavior in the community.
(2) The need for achievement played a negative mediating role between supportive climate and user’s participation behavior, the need for achievement played a suppressing effect between controlling climate and user’s participation behavior; the need for power played a negative mediating role in supportive climate and user’s participation behavior, the need for power played a suppressing effect in supportive climate and user’s citizenship behavior; and the need for affiliation played an mediating role between supportive climate and user’s value co-creation behavior, and similar findings were found in Wann-Yih & Sukoco’s [52] study. These results showed that these three intrinsic motivations can promote the user’s knowledge sharing behavior in OIC.
(3) Community trust had a positive moderating effect between the need for achievement and user’s citizenship behavior, as well as between the need for power and user’s citizenship behavior. The result is in line with the findings of Zhao and Detlor’s (2021) [57] study, indicating that trust plays a moderating effect between the need for power and user’s sharing intensions. This shows that the higher level of community trust strengthens the influence of users' motivation of achievement needs and power needs on their citizenship behavior.

9.The authors should check all the language over the whole manuscript.

**Reply**:

We have carefully checked and improved the English writing in the revised manuscript according to the reviewers' comments. And the manuscript has been revised and re-polished by native English speakers. We hope it will meet the standard of PLOS ONE.

10. In terms of references, due to the fact that empirical research requires a large amount of literature support, the current number of references is relatively small, with at least 60 additional articles with high quality added. Additionally, it is important to ensure that the format meets the requirements of Plos One.

**Reply:**

Thank you very much for your guidance on the Reference List, some of which published in high impact journals. We have added theses important literatures on the Reference List. The number of the references were 64, we hope it will meet the standards of PLOS ONE.

**<3> To Reviewer 2:**

1.Research Motives and Cohesion: The choice of examining user’s participation behavior and user’s citizenship behavior in the context of an online innovation community raises questions. Given the focus on innovation, it might be more pertinent to explore behaviors more closely associated with innovation and creative collaboration. As someone deeply involved in creativity and innovation research, I am keen to understand how your model might relate to innovation and creativity outcomes. It might be beneficial to consider adding this as a sequential outcome variable following the value co-creation behaviors you have tested.

**Reply:**

We are very grateful to you for the valuable advice. According to Jessen et al. (2020), user creativity refers to the ability of users and enterprises to jointly create novel and meaningful products, services, ideas and consumption patterns or experiences. User’s behavior, such as participation behavior and citizenship behavior in online communities, have an impact on creativity in the community (Fan & Fan, 2023). However, the existing literature in online community users' creativity is basically measured by questionnaire data (Fan & Fan, 2023; Jessen et al., 2020), and the data in this paper are measured by objective data. The data about the users' creativity was unavailable, preventing the measurement of the impact of user innovation behavior on creativity.

For future research, we will consider taking the variable of user creativity as a sequential outcome when collecting data through questionnaire, and investigate the influence of online community climate on user’s creativity.

2. Theory of Value Co-Creation: While I am not entirely familiar with the theory of value co-creation you employed; it seems to have a broad application in various business dynamics. I would appreciate it if you could elucidate how this theory specifically relates to the context of an online innovation community. This would provide clarity and strengthen the theoretical foundation of your study.

**Reply：**

As mentioned in the manuscript, value co-creation refers to a process in which enterprises, users and other stakeholders join forces to create value (Alves et al., 2016). In a narrow sense, value co-creation refers to the use value generated by the direct interaction between enterprises and users. In a broad sense, value co-creation is not only the direct interaction between enterprises and users, but also the direct or indirect participation and interaction of users to create value in the process of product design, research and development, production and sales.

Enterprises and users are interdependent, when users interact in the online community founded by enterprises, they can have a strong co-creation relationship with the community and enterprises (Zhao et al., 2019). The value co-creation discussed in this paper refers to the online innovation community situation, in which users directly interact with enterprises and other users, participate in new product research and development, provide feedback, etc., which is beneficial for enterprises to understand users' needs and continuously improve products. The core of value co-creation in online innovation community lies in the process of creating value together in the interaction between users, as well as between users and the communities they engage with.

There have been many articles about online communities and value co-creation, mainly focusing on how to promote users in the community to create value together and the influencing factors of value co-creation. However, there is limited research on how the community climate affects users' value co-creation behavior. Thus, this article mainly investigates the impact of online community climate on users' value co-creation behavior.

3. Choice of User’s Participation Behavior: The decision to use user’s participation behavior over creative engagement is intriguing. I would recommend citing relevant references to justify this choice and provide a clearer rationale for the same.

**Reply**：

According to Yi & Gong (2013), user’s value co-creation behavior can be divided into user’s participation behavior and user’s citizenship behavior. User’s participation behavior refers to the active participation of users in value-creating activities, such as information search, information sharing, responsible behavior, and interpersonal interaction. User’s citizenship behavior refers to the behavior of users to maintain the interests of community users and create additional value spontaneously in the community, such as feedback, help, support and tolerance. We also found similar research classifications were appeared in Bui & Jeng(2022)、Zhao(2019)、and Wang et al.(2021) studies and cited these important literatures in the manuscript.

Thank you again for your positive and constructive comments and suggestions on our manuscript. We tried our best to improve the manuscript and made some changes in the version. We sincerely hope that you will find the revised manuscript is accepted for publication.

Your sincerely,

Qiong Tan, Juan Tan, Xiaohui Gao
