# Supplementary material for: How does the online innovation community climate affect the user’s value co-creation behavior: The mediating role of motivation
Source: PLoS One. 2024 Apr 23;19(4):e0301299. doi: 10.1371/journal.pone.0301299 (PMC11037537; doi:10.1371/journal.pone.0301299)
Supplement: Attachment — Submitted filename: Response to Reviewers.docx [file pone.0301299.s003.docx]

Dear editor,

On behalf of my co-authors, we thank you very much for your letter and for the reviewers’ comments concerning our manuscript entitled “How does the online innovation community climate affect the user’s value co-creation behavior: the mediating role of motivation”. These comments are all valuable and helpful for revising and improving our papers, as well as the important guiding significance to our research. We have studied your comments carefully and have made corrections which we hope meet with approval. The main corrections in the paper and the responds to your letter and the reviewers’ comments are as following.

**<1> To editor:**

1） This study involved 3315 supporters, as I believe informed consent and ethical approval are necessary.

Reply:

Thank you for your prompt feedback and guidance regarding our paper. We appreciate the importance of ethical considerations in research and the need for informed consent.

In response to your concern, we want to show that the study involved 3315 supporters, and while we recognize the importance of informed consent and ethical approval, we would like to explain that the data utilized in our research is freely obtainable from social media platforms, OnePlus community, and is publicly accessible. We want to assure you that throughout our research, we have prioritized data privacy and confidentiality, adhering to all applicable laws and regulations governing data usage and privacy. Thank you for reminding.

Thank you for your understanding, and we remain open to further discussion on any aspects of our study that may require additional clarification.

**<2> To Reviewer:**

1) This paper aims to analyze the existing data but it does not the development of a theory to create a new model. Although the SOR model has been used for your idea, all constructs in the model are not explained in deep-based theory/concept. It depends on types of the data that you collect such the data. Thus, there is still a lack of newness to design the theory development.

Reply：

Thank you sincerely for your valuable insights and feedback regarding the utilization of the SOR model in our paper. In our study, our objective was not to construct an entirely original theory but rather to conduct an in-depth analysis from an integrated perspective. Mehrabian and Russell (1974) extended the SOR model based on environmental psychology, emphasizing that external stimuli (S) influence an individual's cognition, mood, and state (O), subsequently shaping their attitude and behavioral response (R). Drawing upon the SOR framework, we aimed to explore the relationship between online innovation community climate, user motivation and value co-creation behavior.

In this paper, the stimulus factor (S) encapsulates environmental elements, serving as a catalyst for users' perceptual and behavioral changes. The supportive and controlling climates within online communities are integral components of these stimulus factors, directly or indirectly impacting individual responses. The organism factor (O) encapsulates the cognitive and emotional states of individuals, acting as a mediator in the interaction between external stimuli and users' ultimate responses. Reaction (R) is expressed in the form of users' attitude or behavior, and the psychological reaction of users in online communities will ultimately affect their behavior in online communities. Therefore, we put forward the theoretical framework of this paper based on SOR model.

Our research methodology was not a purely data-driven approach; instead, it is grounded in the central question: 'What type of online community climate fosters optimal user participation in value co-creation?' In pursuit of this inquiry, we transposed the Stimuli, Organism, and Response (SOR) theoretical framework, integrating classical motivation theories. This synthesis aims to systematically elucidate the procedural mechanisms underlying the impact of community-supportive climate and controlling climate on users' participation behavior and citizenship behavior, respectively.

This study contributes to the expansion of the application scope of the SOR theoretical framework, enhancing its relevance within the dynamic landscape of online communities. Furthermore, it enriches the application of the SOR theory within the Internet milieu, offering a reinterpretation of the nuanced connotations associated with "S", "O" and "R" in this contemporary context.

We acknowledge the importance of constructing original theories and wish to assure you that it is a direction we are actively pursuing. We greatly appreciate your constructive guidance, and we are committed to enhancing the depth and clarity of our theoretical framework as we continue our research journey.

2) The format of the data scale has not been reported to analyze all factors using SEM because the SEM normally will use the same format of the data scale. The paper uses 7 factors, and 1 modulator, but runs correlation 8 which doesn't make sense because correlation analysis will use only 7 factors for SEM analysis.

Reply:

Thank you sincerely for your insightful observation regarding the number of factors in correlation analysis. As you correctly pointed out, our paper includes 8 factors in the correlation analysis, encompassing 7 primary factors and 1 modulator. We appreciate the opportunity to clarify the rationale behind this approach.

The additional factor introduced in the correlation analysis is rooted in our hypothesis about the potential correlation between the online community climate and community trust. Specifically, we think that a high level of supportive climate within the online community may contribute to an increased level of trust among users. Consequently, we deemed it essential to explore and analyze the correlation between various dimensions of the online community climate, such as supportive climate and controlling climate, and the trust users place in the community.

Furthermore, we draw inspiration from the work of other scholars, for example, Zhang and Bartol’s (Linking empowering leadership and employee creativity: The influence of psychological empowerment, intrinsic motivation, and creative process engagement. The Academy of Management Journal. 2010) study took a similar analytical approach. In their research, the correlation between independent variables and moderating variables was explored, providing a precedent for our analytical methodology.

3) Another test also uses multiple regressions to obtain the results of 15 models using only 5 factors but it does not mention the details e.g. "Why did you select 5 factors?", "How did you use formats of data scale?" etc.

Reply：

Thank you for your insightful observation regarding the testing approach in our paper. In our study, we aimed to explore the moderating effect of community trust on the relationship between user motivation and user value co-creation behavior. User motivation was categorized into three dimensions: Need for Achievement, Need for Affiliation, and Need for Power. Similarly, user value co-creation behavior comprised user participation behavior and user citizenship behavior. Consequently, we anticipated six paths when testing the moderating effect of community trust. However, upon empirical testing, we identified that the path between the need for achievement and user citizenship behavior is not significant. We also draw inspiration from the work of other Journal Q1. In light of this, we focused our analysis on the remaining five paths where significant moderating effects were observed. This adjustment is explicitly mentioned in the paper from lines #478 to #480.

Community trust in our study is defined as users' trust in the community, manifested by their identification with the community. The stronger their identification with the community, the more they believe the community represents their own image, leading to increased trust. To measure community trust, we utilized items related to users' willingness to display personal information within the community.

4) All questionnaires used to collect data have not been reported or clarified. Normally, you should select the questions coming from standards that can be referenced from theories, principles, or research articles that have already been published in Journal Q1. This will help solve the problem of the bias questions.

Reply：

We deeply appreciate your valuable suggestion regarding the data collection process. Your insights on the importance of selecting questions from established standards referenced in theories, principles, or previously published research articles in Journal Q1 are duly noted.

In this paper, the data collection primarily relies on objective data crawled by Python, not questionnaires to measure the variables. In order to collect user data, members of the research group registered as community users and gathered information from the OnePlus community forum. To collect users’ product experience properly, we sampled 14 product sections in the community by Python (as of data collection, a total of 17 sections, excluding three sections beyond the research purpose, including Youleyuan, Qingsheying and Official activities) as the primary source for information collection.

The data required for the research involves both users and products, which were collected in three rounds: in the first round, posts that were officially rated as “hot” in the community were collected, including the popularity value, product section where the post was located, and the link to the personal website. A total of 38,894 posts were collected. In the second round, leveraging the uniqueness of each user's personal website has been obtained in the first round, basic user information was collected, including user's name, ID, number of cheers, displayed items of user information, online time, number of friends, etc. In the third round, the number of comments deleted by the authority, the number of comments on the post, and the number of views on the post collected in the first round were collected. Finally, a total of 676,380 pieces of information were collected.

The inclusion and exclusion criteria are as follows: (1) official activity posts; (2) posts and user information with incomplete information; and (3) only one post randomly included for the same user. As the collection of user information data in this study was related to the posts by users, if all posts by the same user were included, the same user information would be collected repeatedly for users who post multiple times, which would cause problems for data cleaning and sorting. In addition, we randomly deleted multiple posts from the same user to ensure the accuracy of the results. Totally 29,835 pieces of information from 3,315 users were finally included for analysis.

The definitions and measurements of these variables are carefully crafted in alignment with established scholarly definitions and the specific context of the OnePlus community. Furthermore, we have diligently marked the reference scholars in Table 1.

Your feedback has been instrumental in refining our approach, and we are grateful for the guidance provided to enhance the rigor and validity of our research.

5) Path analysis and Hypothesis are not in the same direction or related.

Reply：

Thank you very much for your attention to our research and your feedback on the alignment of path analysis and hypotheses.

We explicitly presented all research hypotheses in Section3, "Research Hypotheses." Following descriptive statistical analysis and correlation analysis, we utilized Mplus8.1 software to test the hypotheses between variables, and we are pleased to report that all indicators fell within the threshold standards. The purpose of the path analysis was to illustrate the relationship between online community climate (supporting climate and controlling climate) and user value co-creation behavior (user’s participation behavior and user’s citizenship behavior). Additionally, we employed the Bootstrap method to test the mediating effect of user motivation. However, we found that some results are inconsistent with the hypothesis.

The findings revealed that the need for achievement exerts a suppressing effect between community climate and user’s participation behavior, the need for power exerts a suppressing effect between supportive climate and user’s value co-creation behavior, and the need for affiliation plays a mediating role between supportive climate and user’s citizenship behavior and user’s participation behavior. We also explained the reasons for the inconsistent results in Page 22, #451-475 in the paper. Finally, hierarchical regression analysis was employed to explore the moderating effect of community trust. The results indicate that community trust positively moderates the relationship between the need for achievement and user’s participation behavior, as well as between the need for power and user’s participation behavior.

We appreciate your valuable feedback and will continue to strive for clarity and comprehensibility in our research. If you have any further suggestions or questions, we would be more than willing to hear them. Thank you very much.

6) Interpretation:
The paper should focus on the overall work "How to get the objective?", "What is the main important point?", "Which issues should focus the work?".
The paper should discuss details such as the implications of theory and practice and the contributions and impacts of your work. It also is hard to explain contributions to the fields for rigorous comparison with alternative methods to find the state-of-the-art. In addition, the paper should have more details of the limitations and future work.

Reply：

We genuinely appreciate your thoughtful insights on our paper, specifically regarding the need for a more focused approach in the implications of theory and practice and the contributions and the limitations.

In response to your constructive feedback, we have diligently incorporated additional details in the sections related to the implications of theory and practice, the contributions of our work as well as the limitations. We trust that these enhancements align with the high standards set by PLOS ONE.

The revised contents are:

**Conclusion and Discussion**

How to promote users' participation in value co-creation in online communities is an increasingly important topic. Based on the SOR model, this study sample included 29,835 pieces of information from 3,315 users in 14 product sections of the OnePlus Community which were analyzed with Mplus8.1, explore the impact of the online community climate on users’ value co-creation behavior in the OnePlus Community and analyzed the mediating role of motivation and the moderating role of community trust. The findings are as follows:

(1) Supportive climate positively affected user’s citizenship behavior, and controlling climate positively affected user’s participation behavior and user’s citizenship behavior. The result is in line with the findings of Zhao et al. [6], whose study showed that supportive climate can promote users' value co-creation behavior by enhancing their psychological capital. Zhu’s (2020) [33] study showed that controlling climate can promote users’ innovation behavior. The stronger the supportive and controlling climate provided by the community, the more it can promote users' information exchange and resource acquisition behavior in the community, thus promoting their value co-creation behavior in the community.
(2) The need for achievement played a negative mediating role between supportive climate and user’s participation behavior, the need for achievement played a suppressing effect between controlling climate and user’s participation behavior; the need for power played a negative mediating role in supportive climate and user’s participation behavior, the need for power played a suppressing effect in supportive climate and user’s citizenship behavior; and the need for affiliation played an mediating role between supportive climate and user’s value co-creation behavior, and similar findings were found in Wann-Yih & Sukoco’s [52] study. These results showed that these three intrinsic motivations can promote the user’s knowledge sharing behavior in OIC.
(3) Community trust had a positive moderating effect between the need for achievement and user’s citizenship behavior, as well as between the need for power and user’s citizenship behavior. The result is in line with the findings of Zhao and Detlor’s (2021) [57] study, indicating that trust plays a moderating effect between the need for power and user’s sharing intensions. This shows that the higher level of community trust strengthens the influence of users' motivation of achievement needs and power needs on their citizenship behavior.

**Theoretical Contribution**

The main theoretical contributions of this study are as follows:

（1）This paper reveals the influence mechanism of online community climate on users’ value co-creation behavior, and expands the related research on user behavior in online community. Earlier studies have shown that the online community climate can have an impact on users’ value co-creation behavior [6], but there is a lack of detailed research on the relationship between the two. In this study, we categorized community climate into supporting climate and controlling climate, users’ value co-creation behavior into user’s citizenship behavior and user’s participation behavior, then we explored the impact of online community climate on these two dimensions, thus promoting the research on ubiquitous value co-creation behavior.

（2）There is a huge body of literature on online community climate and user’s value co-creation behavior [6, 27], but the mechanism whereby the two interact with each other remains unclear. It is found that achievement need motivation plays a suppressing effect between controlling climate and user participation behavior, and power need motivation plays a suppressing effect between supporting climate and user citizenship behavior. Therefore, this study introduced the user’s motivation as a mediating variable, focused on the relationship between online community climate, user’s motivation and user’s value co-creation behavior, and explored the mediating role of the need for achievement, hence enriching the related research on user motivation in online community.

（3）Users’ different level of trust in the community will affect their behaviors in the community. Taking community trust as the moderating variable, the present study explored the moderating effect of community trust between users’ motivation and value co-creation behavior [20], which enriches the research on community trust.

**Implications**

(1) Strengthen the dynamic management of the OIC and maintain the balance between “openness” and “control”. On the one hand, online communities create a positive and supportive climate by putting high-quality posts at the top and giving them different popularity, which makes users in the community more active. On the other hand, it is necessary to properly control the community by various measures, such as controlling community information exchange, strictly reviewing the content of user posts, and stimulating users’ value co-creation behaviors. Community activities are consistent with the innovation goals of enterprises, thereby delivering more valuable innovation results to leading enterprises.

(2) Stimulate the intrinsic motivation of users to participate in value co-creation in the community. The community can improve user loyalty by increasing the user level settings in the community to stimulate their needs for power; at the same time, it can encourage users to make friends in the community, share their views and suggestions with others, meet their needs for affiliation, thus enabling them to have a sense of belonging to the community. The community should get rid of the reward system. In order to get rewards, users are more inclined to engage in easy commenting behaviors [63] rather than actively posting, which is prone to “threadjacking” behaviors [64], making it difficult to manage the community. Through the external stimulation of creating different community climates, online communities can affect the internal motivation of users in the community and transform the external impetus into the internal motivation of personal value co-creation.

(3) Enhance the trust within the community and between community users. On the one hand, the community can encourage community users to speak freely and express some unique and insightful views by creating a good supportive climate, so as to enhance users' perception of the supportive climate in the community. On the other hand, the community can strengthen supervision and control by creating a controlling climate, protect users' personal information, reduce the uncertainty of users' perception of the community, and reduce the risk of participating in community activities to improve the level of community trust.

**Limitations**

First, this study sampled the cross-sectional data of the OnePlus Community product sections for analysis, but the role of community climate may change with time. In the future, longitudinal panel data can be considered to investigate the dynamic changes in the impact of community climate on user behavior. Second, the generality of the conclusions in this study needs to be further verified. We only explored the data in the OnePlus Community and failed to widely adopt product information and user information of other similar industries. Different types of online communities provide different supportive climates and controlling climates, which will affect users’ psychological perceptions of the community and have different impacts on users’ value co-creation behaviors. Third, while the exploration of users’ value co-creation behavior in online innovation communities from the perspective of the community is significant, many issues still need to be explored in depth. More complex models may be used for in-depth research in the future. In addition, this paper only considers the mediating role of achievement motivation and the moderating role of community trust. In the future, we can consider different mediating variables, such as perceived benefits and moderating variables, such as user value concept, and explore the influence mechanism of community climate on user value co-creation behavior.

Thank you again for your positive and constructive comments and suggestions on our manuscript. We tried our best to improve the manuscript and made some changes in the version. We sincerely hope that you will find the revised manuscript is accepted for publication.

Your sincerely,

Qiong Tan, Juan Tan, Xiaohui Gao
