# Supplementary material for: How does the online innovation community climate affect the user’s value co-creation behavior: The mediating role of motivation
Source: PLoS One. 2024 Apr 23;19(4):e0301299. doi: 10.1371/journal.pone.0301299 (PMC11037537; doi:10.1371/journal.pone.0301299)
Supplement: Attachment — Submitted filename: Response to editor.docx [file pone.0301299.s004.docx]

Dear editor,

On behalf of my co-authors, we thank you very much for your letter concerning our manuscript entitled “How does the online innovation community climate affect the user’s value co-creation behavior: the mediating role of motivation”. These comments are all valuable and helpful for revising and improving our papers, as well as the important guiding significance to our research. We have studied your comments carefully and have made corrections which we hope meet with approval. The main corrections in the paper and the responds to your letter is as following.

1> Please note that funding information should not appear in any section or other areas of your manuscript. We will only publish funding information present in the Funding Statement section of the online submission form. Please remove any funding-related text from the manuscript.

**Reply:**

Thank you very much for your guidance on the “funding information”. We have deleted the funding information in the manuscript.

Thank you again for your positive and constructive comments and suggestions on our manuscript. We tried our best to improve the manuscript and made some changes in the version. We sincerely hope that you will find the revised manuscript is accepted for publication.

Your sincerely,

Qiong Tan, Juan Tan, Xiaohui Gao
